# Membranes for Gas Separation and Purification Processes

**DOI:** 10.3390/membranes12060622

**Published:** 2022-06-15

**Authors:** Chong Yang Chuah

**Affiliations:** 1 Department of Chemical Engineering, Universiti Teknologi Petronas, Bandar Seri Iskandar 32610, Perak, Malaysia; 2 CO2 Research Centre (CO2RES), Institute of Contaminant Management, Universiti Teknologi Petronas, Bandar Seri Iskandar 32610, Perak, Malaysia

This Special Issue, entitled “Membranes for Gas Separation and Purification Processes”, was introduced to discuss the recent progress in the development of membranes for gas separation and purification. In general, membranes are capable of improving the gas separation performance as compared to conventional methods such as scrubbing, absorption, cryogenic distillation, and swing adsorption. These existing technologies have been limited by challenges such as a large plant footprint, sophisticated design, and poor energy efficiency. In this regard, membranes have emerged as a promising alternative, and have been utilized in fundamental research and pilot-scale studies. 

One of the most common utilizations of membranes in gas separation involves the study of mixed-matrix membranes (MMMs). In general, MMMs adopt a classical method to allow synergistic improvement in gas separation performance, which is evaluated in terms of gas permeability and selectivity. This is attributed to the presence of nanomaterials, which allows a substantial enhancement in gas permeability and/or selectivity [1]. In this regard, nanomaterials, which are used as filler materials in membranes, are able to effectively tune the gas transport properties of the resulting membrane. For example, carbon dioxide (CO_2_) capture has been a focus of attention, as CO_2_ concentration in the atmosphere has surpassed 400 ppm since 2013 [2]. Therefore, the use of carbon capture and sequestration (CCS) systems is reported to be a feasible solution to minimize the emission of greenhouse gas (GHG) from point sources, namely the combustion of fossil fuels or natural gas [3]. For instance, in the study conducted by Pacheco et al. [4], carbon nanotube (CNT) was proposed as a filler for MMM, to generate a potential improvement in CO_2_ separation performance. CNT, which is a classified as a one-dimensional material with a high aspect ratio, could be used to encourage the preferential transport of CO_2_ against other gases (e.g., nitrogen (N_2_) and methane (CH_4_)) [5]. Due to the larger kinetic diameter of N_2_ and CH_4_ as compared to CO_2_, bulkier gas molecules are forced to adopt a more tortuous path, leading to an increase in the diffusion distance for N_2_ and CH_4_ in the membrane. This eventually results in higher mixed-gas selectivity (e.g., CO_2_/N_2_ and CO_2_/CH_4_) [6]. 

Subsequently, the investigation of MMMs under light hydrocarbon (C_1_–C_3_) separation has been performed. Light hydrocarbons (e.g., methane, acetylene, ethane, propylene, and propane) are major raw materials in petrochemical processes to produce everyday materials (e.g., polyethylene and polypropylene). In terms of unit operation, adsorbents (also known as porous materials) are capable of performing effective separation among light hydrocarbon gases with comparable physical and chemical properties [7]. Nevertheless, swing adsorption, which is critical in this process, suffers from low adsorbate recovery, depending on the type of adsorbents used [8]. Thus, in the case of ethylene/ethane (C_2_H_4_/C_2_H_6_) and propylene/propane (C_3_H_6_/C_3_H_8_) separation, membranes have been proven feasible, as observed in a perspective study on C_2_H_4_/C_2_H_6_ and C_3_H_6_/C_3_H_8_ separation by Chuah et al. [9]. In general, zeolites and metal–organic frameworks (MOFs) have been heavily utilized in this separation process due to their high C_2_H_4_ and C_3_H_6_ gas adsorption performance as compared to C_2_H_6_ and C_3_H_8_, respectively [10]. Particularly, MMMs can feasibly be used to overcome the constructed upper bound curve for C_2_H_4_/C_2_H _6_ and C_3_H_6_/C_3_H_8_ separation, which is critical in advancing the performance of gas separation membranes.

Most of the research performed on MMMs involves polymeric membranes as the polymer matrices, with porous materials incorporated as the filler. On the other hand, composite membrane, which requires the attachment of a molecular sieve layer onto the porous support, can be formed. This creation allows an improvement in mechanical strength as compared to free-standing molecular sieve membranes [11]. Therefore, in the study conducted by Hayakawa et al. [12], zeolite membrane was developed using the rapid thermal processing (RTP) and ozone de-templating methods to prepare aluminum (Al)-containing ZSM-58 zeolite membrane. This approach is able to suppress crack formation as compared to the conventional thermal de-template method, which is utilized to remove the organic structural directing agent during the synthesis of zeolites. Based on the reported data, the ozone de-templating method is able to achieve remarkably high CO_2_/CH_4_ separation performance as compared to the RTP approach. This behavior is attributed to the inability of the RTP process to achieve crack suppression, due to the lack of surface silanol (Si-OH) functionality. On the other hand, Al-containing ZSM-58 membrane is able to suppress the formation of cracks through the RTP approach, which is evident from the increased synthesis time. Nevertheless, with the co-current increase in the thickness of the selective layer, it is anticipated that lower CO_2_ membrane permeance can be achieved. 

Last but not least, the application of membranes in gas sensing and detection was performed by Chen et al. [13]. In this study, membranes for ammonia (NH_3_) gas sensing were utilized alongside penta-graphene (PG), which possesses good dynamical and mechanical stability, up to 1000 K [14]. In particular, based on various theoretical investigations, PG showcases great potential in various applications such as hydrogen storage, gas capture and sensing, and lithium-ion batteries. Thus, the verification of the adsorption structures, gas-sensing properties, and electronic characteristics of pristine and doped (e.g., boron, nitrogen, phosphorous, aluminum and silicon) PG was performed. Based on the calculation, it was observed that pristine PG is insensitive to the toxic gases due to its weak adsorption strength and long adsorption distance. On the other hand, the doping of various atoms allows a transition from the physisorption to chemisorption of NH_3_ into the active sites due to strong orbital hybridization and a large charge transfer between gas molecules and the doped atoms. 

In a nutshell, membranes are able to serve as an appropriate alternative for improved performance in gas separation and purification. Despite the substantial research challenges (e.g., membrane design, membrane configuration and membrane materials) [15] associated with an increase in the practical feasibility of membranes in pilot-scale or industrial applications, it is undeniable that membranes are expected to complement the available conventional process, which suffers from an undesirably large energy penalty and a large plant footprint.
